# Structural Bases for the Synergistic Inhibition of Human Thymidylate Synthase and Ovarian Cancer Cell Growth by Drug Combinations

**DOI:** 10.3390/cancers13092061

**Published:** 2021-04-24

**Authors:** Cecilia Pozzi, Matteo Santucci, Gaetano Marverti, Domenico D’Arca, Lorenzo Tagliazucchi, Stefania Ferrari, Gaia Gozzi, Lorena Losi, Giusy Tassone, Stefano Mangani, Glauco Ponterini, Maria Paola Costi

**Affiliations:** 1 Department of Biotechnology, Chemistry and Pharmacy, Department of Excellence 2018–2022, University of Siena, Via A. Moro 2, 53100 Siena, Italy; pozzi4@unisi.it (C.P.); giusy.tassone@unisi.it (G.T.); stefano.mangani@unisi.it (S.M.); 2 Department of Life Sciences, University of Modena and Reggio Emilia, Via G. Campi 103, 41125 Modena, Italy; matteo.santucci86@gmail.com (M.S.); lorenzotagliazucchi@unimore.it (L.T.); sferrari591@gmail.com (S.F.); g.gozzi@holostem.com (G.G.); lorena.losi@unimore.it (L.L.); 3 Department of Biomedical, Metabolic and Neural Sciences, University of Modena and Reggio Emilia, Via G. Campi 287, 41125 Modena, Italy; gaetano.marverti@unimore.it (G.M.); domenico.darca@unimore.it (D.D.)

**Keywords:** drug combinations, thymidylate synthase, X-ray crystal structures, inhibition kinetics, anticancer agents, synergistic combination

## Abstract

**Simple Summary:**

Drug combinations may help overcome drug resistance, a relevant cause of failure of ovarian cancer therapy. However, designing successful combinations requires a lengthy preclinical validation process. We have analyzed combinations of 5-fluorouracil and raltitrexed, two anticancer drugs that target thymidylate synthase, a key enzyme for the nucleotide synthesis. We have observed administration sequence specific and synergistic combined effects of the two drugs against cisplatin sensitive and resistant ovarian cancer cells. However, the focus of this work was to show that a high stability of the complex of the enzyme with the two drugs, as highlighted by X-ray crystallography, and synergistic inhibition of the enzyme represent indicators, if not prerequisites, for this drug combination to be synergistically active against sensitive and resistant ovarian cancer cells. We thus propose that structural and mechanistic information acquired during the preclinical research can help predict a successful therapeutic application of a drug combination.

**Abstract:**

Combining drugs represent an approach to efficiently prevent and overcome drug resistance and to reduce toxicity; yet it is a highly challenging task, particularly if combinations of inhibitors of the same enzyme target are considered. To show that crystallographic and inhibition kinetic information can provide indicators of cancer cell growth inhibition by combinations of two anti-human thymidylate synthase (hTS) drugs, we obtained the X-ray crystal structure of the hTS:raltitrexed:5-fluorodeoxyuridine monophosphate (FdUMP) complex. Its analysis showed a ternary complex with both molecules strongly bound inside the enzyme catalytic cavity. The synergistic inhibition of hTS and its mechanistic rationale were consistent with the structural analysis. When administered in combination to A2780 and A2780/CP ovarian cancer cells, the two drugs inhibited ovarian cancer cell growth additively/synergistically. Together, these results support the idea that X-ray crystallography can provide structural indicators for designing combinations of hTS (or any other target)-directed drugs to accelerate preclinical research for therapeutic application.

## 1. Introduction

Drug combination represents an important therapeutic achievement in the anticancer clinical practice [1]. Because anticancer drugs show low therapeutic indexes with a high risk of toxicity and side effects, empirical validation of the effects of a drug combination can only be demonstrated through a lengthy and costly investigational path, i.e., first in a preclinical cellular study, then in animal models, and, finally, in a clinical trial [2]. Thus, an early indicator of the efficacy of a drug combination is desirable, yet highly challenging. In the present work, we show how analysis of co-crystal structures of human thymidylate synthase (hTS) with two drugs bound within a single catalytic cavity in distinct, proximal binding sites can provide molecular indicators of synergistic inhibition of cancer cell growth by the two drugs. We selected two widely used drugs, 5-fluorodeoxyuridine monophosphate (FdUMP), the bioactive metabolite of the 5-fluorouracil (5FU), and raltitrexed (RTX, N-[(5-{methyl[(2-methyl-4-oxo-1,4-dihydroquinazolin-6-yl)methyl] amino}-2thienyl) carbonyl]-L-glutamic acid). 5FU and RTX are both used in chemotherapy against solid cancers [3]. They inhibit hTS by targeting the enzyme active site. While FdUMP competes with the substrate, dUMP (2′-deoxyuridine-5′-monophosphate), RTX competes with the cofactor, N^5^, N^10^-methylenetatrahydrofolate (mTHF). RTX, a classical folate antimetabolite and a selective TS inhibitor, shows a mixed-type inhibition mechanism [4]. Because of their different binding sites, FdUMP and RTX might not hinder each other and, in the absence of adverse protein conformational changes, might bind simultaneously to an enzyme molecule, and exhibit synergistic overall inhibitory activity. Preclinical data suggest that RTX may be successfully combined with 5FU to improve the cure of advanced colorectal cancer (CRC) [5] and head and neck squamous cell cancer [4]. These studies also highlighted the schedule-dependent synergism between RTX and 5FU, prompting clinical studies of this combination [6]. According to preclinical data, the concurrent administration of the two drugs or the administration of RTX followed by 5FU were more successful than other schedules [7]. However, these cell-based experiments were not supported by a molecular mechanistic explanation of the reported observations.

Taking advantage of a large quantity of experimental data available, we have adopted a non-conventional approach, amounting to a detailed analysis of how FdUMP and RTX can interact with the hTS active site, to clarify the molecular basis of the combination of these two drugs. We have first obtained the X-ray crystal structure of the hTS:FdUMP:RTX ternary complex; then, we have investigated the mechanistic features of the synergistic inhibition of the recombinant enzyme by the two compounds; finally, we have explored the efficacy of combinations of RTX and 5FU administered to a panel of ovarian cancer cell lines according to different sequences and have characterized the combination patterns. Our study supports the concept that X-ray crystallography can be an indicator of the synergistic effect observed on cancer cells. In this work, we outline conditions that must be fulfilled for this approach to be useful against other targets.

## 2. Materials and Methods

### 2.1. Drugs and Chemicals

RTX was purchased from Selleckchem (Houston, TX, USA) and was dissolved in DMSO shortly before its addition to cell cultures. Cells treated with the vehicle only (DMSO, maximum concentration, 0.5% (*v/v*) in media) served as control. All other chemicals were purchased from Sigma-Aldrich S.r.l. (Milan, Italy), except otherwise indicated.

### 2.2. Protein Cloning and Purification

The human TS protein was produced as His^6^-tag protein (the non-cleavable N-terminal His^6^-tag was encoded by the pQE80L expression plasmid) in the *E. coli* strain BL21(DE3) as previously described [8].

### 2.3. Crystallization and Structure Determination of the hTS:FdUMP:RTX Ternary Complex

Crystals of the hTS:FdUMP:RTX ternary complex were obtained by co-crystallization in a hanging-drop setup [9] at 293 K. Drops were prepared by mixing equal volumes (2 μL) of the ternary complex (hTS, 5.7 mg/mL, in 100 mM HEPES buffer, pH 7.5, containing a 10-fold molar excess, ~ 1.5 mM, of both inhibitors) and precipitant solution (25% *w/v* PEG4000, 30 mM ammonium sulfate, and 20 mM β-ME in 100 mM TRIS buffer, pH 9). Crystals of the ternary complex appeared in ~ 2 weeks in drops equilibrated over 600 μL of precipitant solution and grew to a final size of ~ 0.05 × 0.1 × 0.1 mm^3^ in about 6 weeks. Crystals were later transferred in a cryoprotecting solution consisting of the precipitant solution with added 15% (*v/v*) ethylene glycol, and flash-frozen in liquid nitrogen.

Diffraction data were collected at 100 K at the European Synchrotron Radiation Facility (ESRF) beamline ID23-2. Data collection parameters are reported in Table S1 of the Supplementary Information. Data were indexed and integrated with the program Mosflm [10] and scaled with Scala [11,12] from the CCP4 suite [13]. The structure was solved by molecular replacement in space group P1 using MOLREP [14] from the CCP4 suite [13]. The starting model consisted of native hTS determined in the active conformation (PDB: 1HVY [15], excluding solvent molecules and non-protein atoms). The molecular replacement solution obtained in space group P1 consists of three independent hTS dimers where each of the six subunits has both FdUMP and RTX bound. The model of the complete structure has been refined to full convergence in space group P1 using Refmac5 [16]. The refinement protocol consisted of several cycles of refinement alternated with manual rebuilding and checking of the model. Non-crystallographic symmetry was used in the first cycles of refinement to increase the data/parameter ratio and was released in the last stages to allow the identification of differences among the different subunits. Due to the medium resolution of the structure, only 376 water molecules could be confidently located and added to the model in the final stages by using the program Coot [17,18]. For the same reason, disordered loops and several side chains could not be visualized in the electron density maps and were omitted from the model. The stereochemical quality of the final model was checked using Coot and Procheck [17,18,19]. Data reduction and refinement statistics are reported in Tables S1 and S2 of the Supplementary Information. Coordinates and structure factor for the complex hTS:FdUMP:RTX have been deposited in the PDB under the accession code 6ZXO.

### 2.4. Enzyme Kinetics and Inhibition Assays

The hTS kinetic parameters (k_cat_ and K_m_) were determined spectrophotometrically using a Beckman Coulter DU-640 UV-vis spectrophotometer. The method consists of measuring the absorbance increases at 340 nm due to dihydrofolate (DHF) formed by oxidation of mTHF with time. The reaction was carried out at pH 7.0 in 600 µL of a buffer including 30 mM NaCl and 20 mM NaH_2_PO_4_, and 50% (*v/v*) of TES buffer (100 mM N-[tris(hydroxymethyl)methyl]-2-aminoethanesulphonic acid, 50 mM MgCl_2_, 13 mM formalin, 2 mM EDTA, 150 mM β-ME, pH 7.4), 0.3 µM hTS enzyme, and 55 µM mTHF. The reaction was started by adding dUMP at a 120 μM final concentration. The k_cat_ and K_m_ values were determined according to the classical Michaelis–Menten kinetics. K_m_ for dUMP was 10 μM and K_m_ for mTHF was 5 μM.

The inhibition of the hTS activity by RTX and FdUMP, both individually and in combination, was measured by a spectrophotometric assay on a 96-well plate using a Molecular Device Spectramax-190 multiplate reader. Each kinetic run consisted in monitoring the absorbance at 340 nm for 180 s at room temperature. The reaction mixture contained, added in this order, the kinetic buffer (30 mM NaCl, 20 mM NaH_2_PO_4_, pH 7.0), 50% TES buffer, the hTS enzyme, at a final concentration of 300 nM, the mTHF cofactor (55 μM), and, to start the enzymatic reaction, dUMP (120 µM). First, we performed experiments with RTX and FdUMP alone at six concentrations between 0 and 400 nM for RTX and 0 and 1600 nM for FdUMP. Then, in order to test the effect of combinations of the two drugs, the RTX inhibition activity was tested at varying concentrations in the 0.024–1 µM range in the presence of a fixed concentration of FdUMP, 450 nM. Conversely, we measured the inhibition by FdUMP at concentrations between 0.238 and 10 µM in the presence of 120 nM RTX. For each tested inhibitor concentration, or concentration combination, we measured three replicates. The results of single-inhibitor experiments were analyzed within the tight-binding inhibition approach, described in the Supplementary Information. The results of combined inhibition experiments were semi-quantitatively analyzed using Dixon-type plots and isobolograms [20].

### 2.5. Cell Lines

The human ovarian cancer cell lines, A2780, A2780/CP, 2008, C13*, and IGROV-1, were grown as monolayers in RPMI 1640 medium containing 10% heat-inactivated fetal bovine serum and 50 µg/mL gentamycin sulfate. The 2008 cell line was established from a patient with serous cystadenocarcinoma of the ovary [21]. The cisplatin (cDDP)-resistant variant C13* cells derived from the parent 2008 cell line, are about 13-fold resistant to cDDP and were developed by monthly exposure to cDDP, followed by chronic exposure to stepwise increases in cDDP concentration [22]. The human ovarian carcinoma A2780/CP cells are about 10-fold resistant to cDDP and derived from the parent A2780 cell line [23]. The IGROV-1 cell line, originating from an ovarian carcinoma of a 47-year-old woman, was established in monolayer tissue culture. The IGROV-1 cell line exhibits an epithelial character, highly tumorigenic properties and a low doubling time. In addition, some cytogenetic markers contain oncogenic rearrangements [23]. All cell media and the serum were purchased from Lonza (Verviers, Belgium). Cultures were equilibrated with humidified 5% CO_2_ in air at 37 °C. All studies were performed in Mycoplasma negative cells, as routinely determined with the MycoAlert Mycoplasma detection kit (Lonza, Walkersville, MD, USA). The protein content in the various assays was estimated by Lowry’s method [24] unless otherwise indicated.

### 2.6. Cell Growth Assay

The experimental schedules to determine the optimum conditions for combining 5FU and RTX included: (a) simultaneous addition of 5FU and RTX, then 72 h incubation, (b) sequential exposure to 5FU for 24 h, then to RTX for a further 48 h, and (c) the interchanged schedule, RTX for 24 h and then 5FU for a further 48 h. Cell growth was determined using a modified crystal violet assay [25]. At the scheduled times, the tissue culture medium was removed, and the cell monolayer was fixed with methanol and stained with 0.2% crystal violet solution in 20% methanol for at least 30 min. After being washed several times with distilled water to remove excess dye, the cells were left to dry. The incorporated dye was solubilized in acidified isopropanol (1N HCl: 2-propanol, 1:10 *v/v*). After appropriate dilution, the absorbance was measured at 540 nm. The concentration of extracted dye was proportional to the cell number. The percentage of cytotoxicity was calculated by comparing the absorbance of cultures exposed to the drug to that of unexposed (control) cultures.

### 2.7. Biological Synergy Analysis

The effects of drug combination were quantified by the synergism quotient [26,27,28]. This is defined as the net growth inhibitory effect of the drug combination divided by the sum of the net individual effects. A quotient larger than unity indicates a synergistic effect, while a quotient lower than unity indicates an antagonistic effect. The heatmap and clustering were realized with the open-source software R (RStudio, http://www.rstudio.com/, accessed on 7 September 2020) and Bioconductor (Bioconductor, https://www.bioconductor.org/, accessed on 7 September 2020) repository, using ggplot2 and Heatplus packages (https://cran.r-project.org/; https://www.bioconductor.org/, accessed on 7 September 2020). For the clustering analysis, in order to highlight the distance between antagonism, addition and synergy values, the synergism quotient values were elaborated as follows: for synergism quotient values < 0.9, a value of 10 was subtracted; for synergism quotient values ≥ 1.1, a value of 10 was added.

### 2.8. Cell Cycle Analysis

Twenty-four hours after seeding, cells were exposed to the three combination sequences tested for cell growth assay: simultaneous drug exposure to 5FU or RTX for 3 days; exposure to 5FU for 24 h, then to RTX for a further 48 h; RTX for 24 h and then 5FU for a further 48 h. At the scheduled times, the tissue culture medium was removed, and the quantitative measurements of the cell cycle phase distribution were performed by flow cytometry [29]. Briefly, cells were incubated with 10 mM 5-bromodeoxyuridine for 1 h at 37 °C and labelled with monoclonal anti-5-bromodeoxyuridine (Clone MoBu-1, Sigma) in conjunction with a goat antimouse IgG-FICT (Fab*specific, Sigma). Cells were then suspended in 0.5 mL of hypotonic fluorochrome solution (50 µg/mL PI, 0.1% sodium citrate, 0.1% Triton X-100). The samples were kept at 4 °C in the dark for at least 30 min, dispersed by repeated pipetting before flow cytometric analysis in a FACS Coulter Epics XL flow cytometer equipped with a single 488 nm argon laser. The percentages of nuclei in the different phases of the cell cycle (G0/G1, S and G2/M) were calculated using a DNA cell cycle analysis software (Cell-Fit, Becton Dickinson). A minimum of 10^4^ cells were analyzed for each sample.

### 2.9. Statistical Analysis

All values reported are the mean ± SEM unless otherwise indicated. Statistical significance was estimated by a two-tailed Student’s *t*-test performed using the Microsoft Excel software; a difference was considered significant at * *p* < 0.05 or ** *p* < 0.01.

## 3. Results

### 3.1. Crystal Structure of the hTS:FdUMP:RTX Ternary Complex

**Figure 1:**
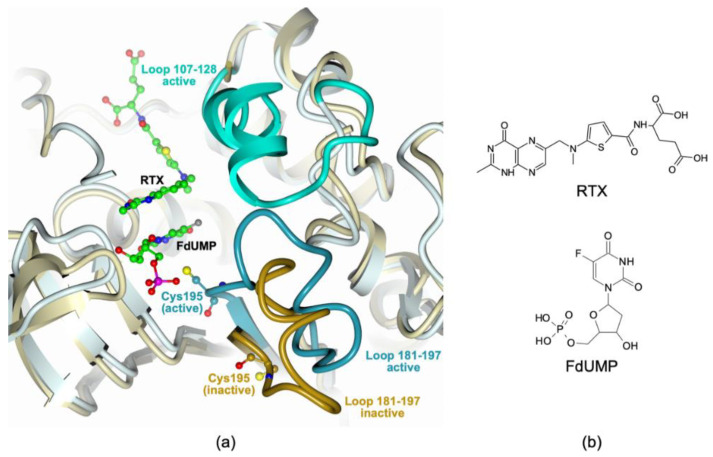
(**a**) Least-squares superposition of hTS in the active (light cyan ribbons; hTS:FdUMP:RTX complex, PDB id: 6ZXO) and inactive (light gold ribbons, PDB id: 3N5G [33]) conformations. The structured loop (residues 181–197) is shown as either a dark cyan or a dark gold rope for the active and inactive conformer, respectively. The different orientations of the catalytic Cys195 residues, represented as balls and sticks, can be appreciated. When the 181–197 loop is in the active conformation, loop 107–128 is structured (cyan rope) whereas it is disordered, thus not visible, when loop 181–197 is in the inactive conformation. The FdUMP and RTX molecules, bound to the “closed” active site, are represented as balls and sticks (green carbons). Oxygen atoms are colored red, nitrogen blue, sulfur yellow, phosphorous magenta, and fluorine gray. (**b**) Chemical structures of RTX and FdUMP.

The X-ray crystal structure of the hTS:FdUMP:RTX ternary complex will be described in detail in comparison with the existing complexes of the individual inhibitors. Human thymidylate synthase adopts two different conformations, named active and inactive, that equilibrate with each other [30]. The equilibrium position depends on mutations [31,32], on binding of substrates/substrate analogs/inhibitors either in the active site cavity or at the dimer interface, and on crystallization conditions [15,32,33,34]. Figure 1 shows a comparison of two hTS protomers in the active and inactive conformations.

In the latter, the loop formed by residues 181–197 adopts a conformation in which the catalytic Cys195 lies out of the active site (dark gold in Figure 1). When the 181–197 loop is in the inactive conformation, loop 107–128 is normally disordered and never visible in the electron density maps. When the 181–197 loop is in the active conformation (dark cyan in Figure 1), with Cys195 pointing into the active site, the 107–128 loop (cyan in Figure 1) becomes ordered and fully visible in the maps. The present crystal structure of the hTS:FdUMP:RTX complex shows six independent enzyme subunits arranged in three homodimers in the triclinic unit cell. All six subunits are in the active conformation and host in the active site one FdUMP and one RTX molecule. All of them display the same overall structure with RMSDs on the 289 Cα atoms ranging between 0.38 and 0.46 Å.

**Figure 2:**
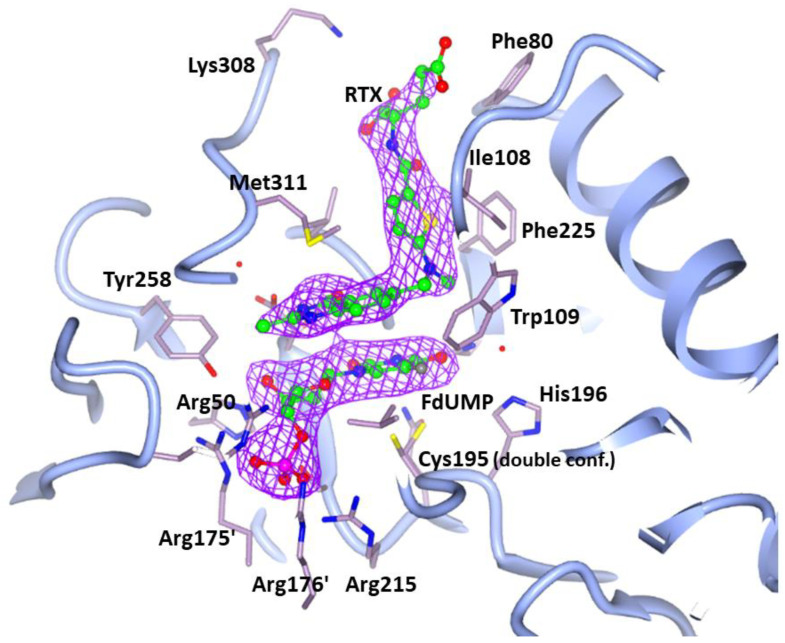
Unbiased omit map (purple mesh) contoured at 3.5 σ, showing the two inhibitors FdUMP and RTX (balls and sticks, green carbons) bound to the hTS active site (light blue cartoon, residues in sticks, lilac carbons) and their positions with respect to Cys195 (modeled in two alternate conformations, one pointing towards the pyrimidine ring and the other one pointing towards the opposite direction, both not bound to FdUMP) and the other key neighboring residues within the enzyme catalytic cavity, such as Trp109. Nitrogen, oxygen, and sulfur atoms are shown in the conventional blue, red, and yellow colors. Phosphorus and fluorine atoms are displayed as magenta and gray spheres, respectively.

**Figure 3:**
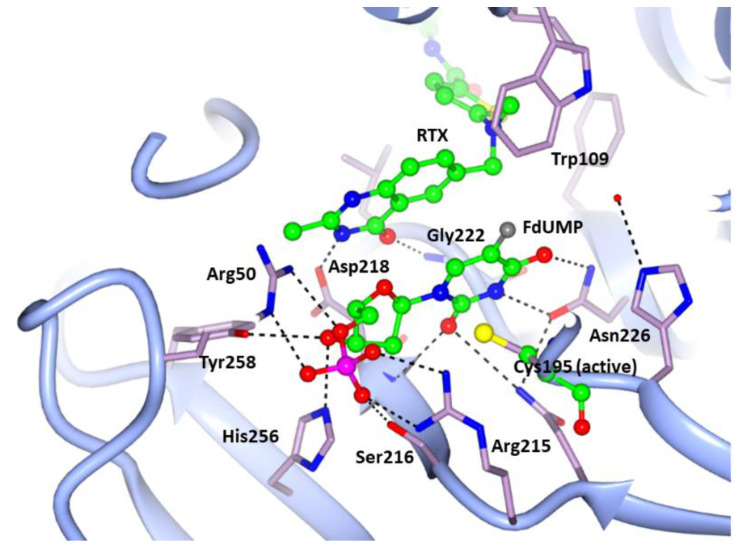
Interactions of FdUMP and RTX (balls and sticks, green carbons) within the active site cavity of hTS (light blue cartoon, residues in sticks, lilac carbons), as they occur in subunit C (Arg175′ and Arg176′ are omitted for clarity). Dashed lines represent H-bonds occurring between the inhibitors and the enzyme. Nitrogen, oxygen, and sulfur atoms are shown in the conventional blue, red, and yellow colors. Phosphorus and fluorine atoms are displayed as magenta and gray spheres, respectively.

Figure 2 provides a view of the active site of an hTS protomer and shows the electron density of the bound FdUMP and RTX molecules. Figure 3 shows in detail the interactions of the two drugs with enzyme residues.

FdUMP and RTX bind in the same sites and with the same orientations found for dUMP and RTX in the hTS:dUMP:RTX ternary complex structures (PDB: 1HVY [15], 1I00 [32], 5X5Q [35]). In particular, FdUMP occupies the same binding site as the natural substrate, dUMP, and is at contact distance with the catalytic Cys195. In our complex, the Sγ of Cys195 does not establish a covalent bond with the C6 of FdUMP, at variance with what is observed for dUMP in the hTS:dUMP:RTX 1HVY structure [15]. As shown in Figure 2 and, in more detail, in Figure 3, stacking interactions occur between the FdUMP pyrimidine ring and the quinazoline ring of RTX and contribute to the stability of the complex. Similar interactions were also found in the 1HVY and 1I00 structures of the hTS:dUMP:RTX ternary complex [15,32]. On the other hand, in our hTS:FdUMP:RTX structure, the fluorine atom of FdUMP establishes a strong non-covalent interaction (3.5(2) Å) with the aromatic side chain of Trp109 that defines a wall of the active site (Figure 2 and Figure 3). The occurrence of such an interaction is confirmed by the larger distances found between the dUMP C5 atom and Trp109 in the hTS:dUMP:RTX ternary complex, where the disordered side chain of Trp109 is modeled in two different orientations, with these distances ranging between 4.1 Å and 7.1 Å in structure 1I00 and between 4.3 Å and 6.4 Å in structure 5X5Q [32,35]. A long F-Trp109 distance, from 5.8 Å to 6.1 Å, is also observed in the hTS:FdUMP binary complex (PDB: 6QXG [36]), in which the side chain of Trp109 is rotated by ~ 43° away from the inhibitor with respect to the orientation observed for the same residue in the ternary complex with RTX. On the other hand, the same short-distance interaction between the FdUMP fluorine atom and the Trp103 side chain is observed in the ternary complex of mouse TS with FdUMP and 5-methyl-tetrahydrofolic acid (PDB: 5FCT [37], Trp103 of mouse TS matches Trp109 of the human enzyme). Leaving out the Cys195 Sγ – dUMP-C6 covalent bond observed in the 1HVY structure [15], the RMSDs between FdUMP and dUMP poses within the hTS active site are within the experimental error (0.5–0.8 Å < 3σ on atomic positions) in all four structures of the ternary complexes.

In the hTS:dUMP/FdUMP:RTX ternary complexes, the phosphate group of FdUMP is bound in the same way as the dUMP phosphate; RMSD upon superimposition of the two structures showed only minor changes at the interface of the two subunits where it engages salt links with four arginine residues belonging to both dimer halves (Arg50 and Arg250 from one subunit and Arg175′ and Arg176′ from the facing protomer), and a H-bond with the side chain of Ser216. The ribose hydroxyl of FdUMP engages two H-bonds with His256 Nε2 and Tyr258 OH (Figure 3).

A double H-bond bridge, reminiscent of the nucleic acid base pairs, links the FdUMP pyrimidine N3 and O4 atoms to Asn226 Nδ2 and Oδ1, respectively. The pyrimidine O2 atom is H-bonded to the backbone NH of Asp218. The fluorine atom of FdUMP is within contact distance (3.6–4.0 Å) from the side chain of Trp109 while it does not interact with the side chain of the catalytic Cys195 that points away from it (Figure 3). These interactions are conserved in all six independent hTS:FdUMP:RTX subunits.

RTX, besides giving the mentioned stacking interactions with FdUMP, is involved in few H-bonds with the enzyme: the quinazoline N3 and O4 atoms are H-bonded to the Asp218 side chain and to the NH of Gly222 respectively (Figure 3). The RTX terminal glutamic acid displays weak electron density and appears disordered and in different conformations. In some subunits (chains C, D, and E in our model), the terminal RTX carboxylate establishes two H-bonds with the NH and CO backbone groups of Met309. Contrarily to the present and the 1I00 and 1HVY structures, the RTX molecule of structure 5X5Q has been modeled with the quinazoline N1 H-bonded to the Asp218 side chain, while the 4-oxo group of this ring points away from it [15,32,35]. When native hTS is in the inactive conformation all cysteine residues but the buried Cys210 react with β-mercaptoethanol (β-ME) present in the crystallization solution [33]. On the contrary, in the hTS:FdUMP:RTX complex, none of the five Cys residues have been modified by β-ME. In summary, in our hTS:FdUMP:RTX complex the two inhibitors, simultaneously bound to the hTS catalytic pocket, establish non-covalent interactions that stabilize the ternary complex. The resulting stability of this assembly, with the two ligands interacting with each other in well-ordered orientations, lends credibility to the expectation that they can act synergistically versus this enzyme.

### 3.2. Synergistic Inhibition of the hTS Enzyme by FdUMP/RTX Combinations

**Figure 4:**
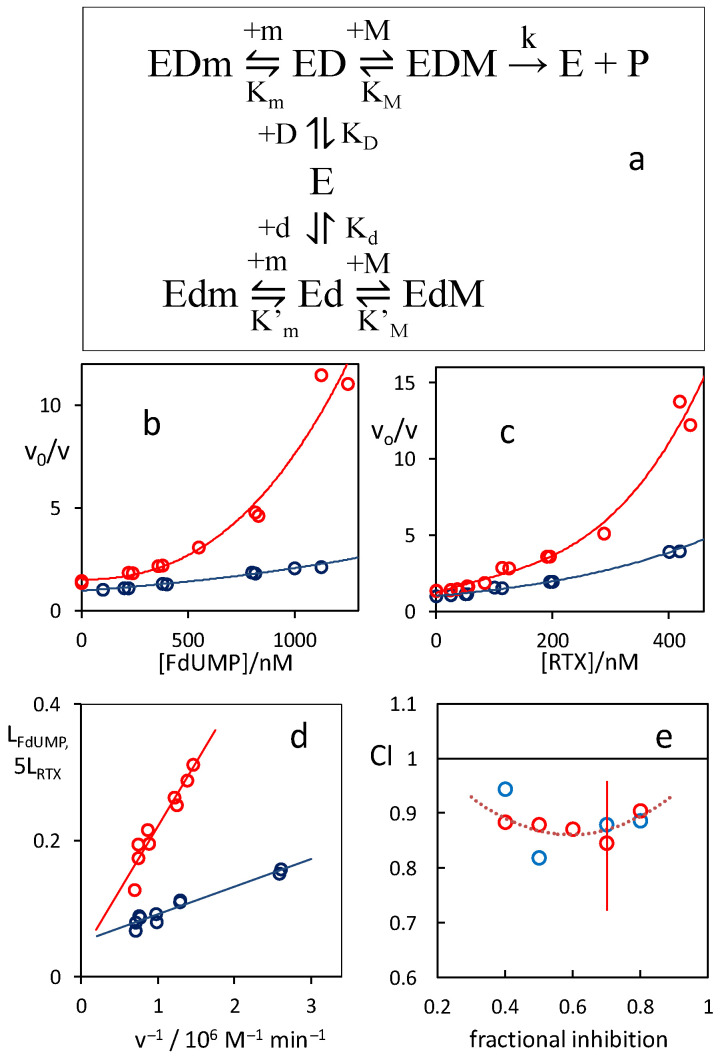
(**a**) Kinetic scheme for the inhibition of hTS (E) by FdUMP (**d**) and RTX (m); D represents the dUMP substrate, M the mTHF cofactor; (**b**,**c**) blue symbols: single-inhibitor Dixon-type plots for FdUMP (**b**) and RTX (**c**) alone; red symbols: Dixon-type plots for the inhibition by combinations of FdUMP with RTX 120 nM (**b**) and of RTX with FdUMP 450 nM (**c**). v_o_ (rate without inhibitors) = 16 μM min^−1^. The curves only represent exponential-function fittings of the data. (**d**) Tight-binding analysis of the single-compound inhibition data of hTS by FdUMP (red symbols, L_FdUMP_) and RTX (blue symbols, 5 L_RTX_). v is the reaction rate, L_FdUMP_ and L_RTX_ are the left members in Equations S1 and S2 in the Supplementary Information. The values of L_FdUMP_ reported in the plot were calculated assuming K_M_’ = 5 K_M_. (**e**) Isobologram showing the combination indexes (CI, see the text) for RTX/FdUMP combinations giving the fractional inhibitions (fi = 1 − *v/v*_0_) reported. Blue circles: [FdUMP] = 450 nM, [RTX]/nM = 55 (fi = 0.4), 70 (fi = 0.5), 120 (fi = 0.6), 180 (fi = 0.7), 260 (fi = 0.8); red circles: [RTX] = 120 nM, [FdUMP]/nM = 100 (fi = 0.4), 300 (fi = 0.5), 450 (fi = 0.6), 650 (fi = 0.7), 1000 (fi = 0.8). The curve is just a quadratic-function fitting to the CI/fract.inhib. data points. A typical estimated error bar is shown.

In order to obtain reference values to which the activities of the combinations could be compared, we first measured the inhibitions of hTS by RTX and FdUMP alone. Because of the high affinities/activities, the inhibitor concentrations were lower than or comparable with the enzyme concentration. Therefore, at variance with what is usually assumed within the Michaelis–Menten (MM) approach, depletion of free inhibitor due to binding to the enzyme could not be neglected. Indeed, standard Dixon plots were curved (blue symbols in Figure 4b,c) thus proving this assumption to be inadequate to account for the single-inhibitor results.

A kinetic scheme for the inhibition of hTS by two analogs (d and m) that bind in the catalytic pocket at the binding sites of the corresponding substrate and cofactor is shown in Figure 4a. The substrate, dUMP (D), and the cofactor, mTHF (M), bind sequentially at their sites in the catalytic pocket [38]. The kinetic model includes the possibility that the two inhibitors bind simultaneously, i.e., that they are not mutually exclusive (complex Edm). We have analyzed the inhibition of hTS by the two compounds alone according to the scheme in Figure 4a using a kinetic data analysis for tight inhibitor binding [20]. Details of these analyses and the equations obtained are given in the Supplementary Information. The data were fairly well described (Figure 4d). In both cases, the catalytic rate constant (k) values obtained from the least-squares intercepts (80 ± 30 min^−1^) were consistent with the reported k values of hTS. For the dissociation of RTX from the hTS:dUMP:RTX (EDm) complex, we obtained an equilibrium constant K_m_ = 8 ± 4 nM. On the other hand, for the dissociation of FdUMP from its complex with hTS (Ed), the K_d_ value obtained depends on the assumption of the unknown value of K_M_’, the dissociation equilibrium constant for the binding of the mTHF cofactor (M) to the hTS:FdUMP complex (Ed). We obtained K_d_ = 164 nM if we assumed K_M_’ = 0.5 K_M_, 86 nM if K_M_’ = K_M_, 47 nM if K_M_’ = 2 K_M_, 23 nM if K_M_’ = 5 K_M_, and 15.6 nM if K_M_’ = 10 K_M_.

We now turn to the analysis of hTS inhibition by combinations of FdUMP and RTX administered concurrently. A comparison between the Dixon-type inhibition plots for FdUMP alone and in combination with 120 nM RTX (red symbols in Figure 4b) and for RTX alone and in combination with 450 nM FdUMP (red symbols in Figure 4c) shows that, while both plots are curved for the reason given previously, the tangents at the origin of the two-inhibitor plots are larger than those of the corresponding single-inhibitor plots, a finding expected for mutually non-exclusive ligands that give synergistic inhibition [20]. From exponential fittings of the Dixon plots in Figure 4b,c in the 0–100 nM abscissa range, we compute average ratios of the slopes of the tangents at the origin of the red (combination) to the blue (single inhibitor) curves 3 ± 0.2 and 2.2 ± 0.2, respectively.

A more explicit demonstration of the synergism of the two inhibitors is provided by the isobologram in Figure 4e. This kind of analysis is based on the observable combination index, CI = D_AC_/D_A_ + D_BC_/D_B_, where D_A_ and D_B_ are the concentrations of inhibitors A and B that produce a given fractional inhibition when administered alone and D_AC_ and D_BC_ the concentrations of the two inhibitors that produce the same fractional inhibition when combined. A simply additive effect would correspond to a CI value of 1. Values lower than 1, as shown in Figure 4e for combinations of RTX and FdUMP versus hTS, indicate a synergistic effect [20]. This purely empirical analysis does not assume any mechanistic view. However, it points to synergism and, together with the Dixon-type plots in Figure 4b,c, supports the conclusion that these two inhibitors are mutually non-exclusive and, in agreement with the X-ray structural evidence, demonstrates that they bind at different sites on the enzyme without hampering each other. We now show that this is a necessary condition for two substrate analogs of hTS to act synergistically towards the enzyme.

### 3.3. Mechanistic Bases of the Synergism for Two Active-Site TS Inhibitors

**Figure 5:**
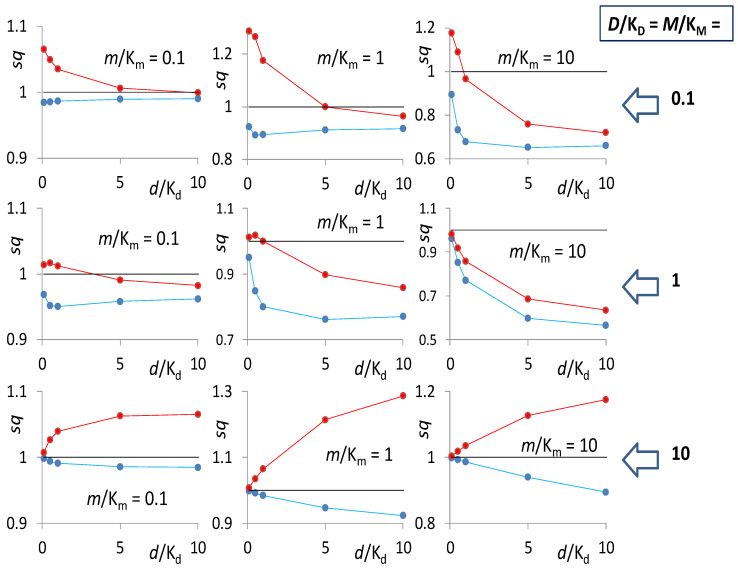
Synergism quotients (*sq*) calculated for the kinetic scheme in Figure 4a in the cases of simultaneous binding of the two inhibitors (m and d, red symbols) and in the case of mutual exclusivity of the inhibitors (blue symbols) for several choices of *i*/K_i_ ratios (from the top, 0.1, 1, and 10). The black line separates the synergistic (*sq* > 1) from the antagonistic values (*sq* < 1).

We have solved the kinetic scheme in Figure 4a for simultaneous inhibition by FdUMP (d) and RTX (m) under the classical MM assumptions, i.e., equilibrated reactions and substrate and inhibitor concentrations larger than the enzyme concentration. Because our inhibitors are tight binders of hTS, such a model can only offer an approximated description of our case. For the reaction rates with either inhibitor alone, *v*_d,m_ = *v*_i,_ and with the two combined, *v*_dm_, we obtained the expressions in Figure S1 of the Supplementary Information. From these expressions, we calculated the synergism quotient, (*sq*) defined as the ratio of the decrease in reaction rate produced by the two inhibitors together at concentrations [d] ≡ *d* and [m] ≡ *m*, to the sum of the decreases measured with the two inhibitors alone at the same concentrations: *sq* = (*v_ni_ − v_dm_*)/(2*v_ni_ − v_d_ − v_m_*), *v_ni_* being the reaction rate in the absence of inhibitors. This descriptor depends on the values of *i*/K*_i_*, i.e., the ratios of the concentrations of each substrate, i = D (dUMP) and M (mTHF), and each inhibitor, i = d (FdUMP) and m (RTX), to the dissociation constant for the corresponding complex with the enzyme (K_D_, K_d_), or with the ED (K_M_, K_m_) and the Ed (K_M_’, K_m_’) binary complexes. The expression for *sq* in terms of the six values of *i*/K*_i_* is reported in Figure S1. We have explored this dependence assuming either the mutually exclusive binding of the two inhibitors, i.e., K_m_’» K_m_ and negligible concentration of the Edm complex, or the simultaneous binding (K_m_’ ~ K_m_). In Figure 5, we show the *sq* values, obtained under these two mechanistic assumptions, for some representative *i*/K_i_ ratios. Results obtained exploring broader ranges of *i*/K_i_ ratios are plotted in Figures S2–S5. As a general result, for any combination of the *i*/K_i_ ratios, the *sq* values obtained in the case of simultaneous binding of the two inhibitors (red symbols in Figure 5 and Figures S2–S5) are larger than those obtained in the mutually exclusive binding case (blue symbols). The mutually exclusive binding is always antagonistic, i.e., the calculated *sq* values are always lower than unity, the value that corresponds to additivity of the effects. On the other hand, if the two inhibitors are allowed to simultaneously bind the enzyme, the *sq* takes quite broadly varying values, from far below to largely above unity, i.e., from antagonism to synergism, depending on the combinations of *i*/K_i_ ratios. Generally speaking, a K_M_’ larger than K_M_, i.e., a lower affinity of mTHF (M) for the hTS:FdUMP (Ed) complex than for the hTS:dUMP (ED), favors synergism. The same is true, quite obviously, for a decrease in the value of K_m_’, i.e., a stabilization of the Edm ternary complex. The extent of these differences, however, are subtly dependent on the values of the *D*/K_D,_ *M*/K_M_, *d*/K_d_, and *m*/K_m_ ratios.

Because of the approximated and simplified character of our analysis, we will not linger on a detailed report of the results obtained, in terms of synergism, with many different *i*/K_i_ combination choices. We only conclude that, according to our kinetic model, synergistic inhibition of recombinant hTS requires binding of RTX to the hTS:FdUMP complex with an affinity comparable with or better than that of RTX for the hTS:dUMP complex (K_m_’ ≤ K_m_).

### 3.4. Inhibitory Effects of RTX and 5FU Combinations on the Growth of Ovarian Cancer Cells

**Figure 6:**
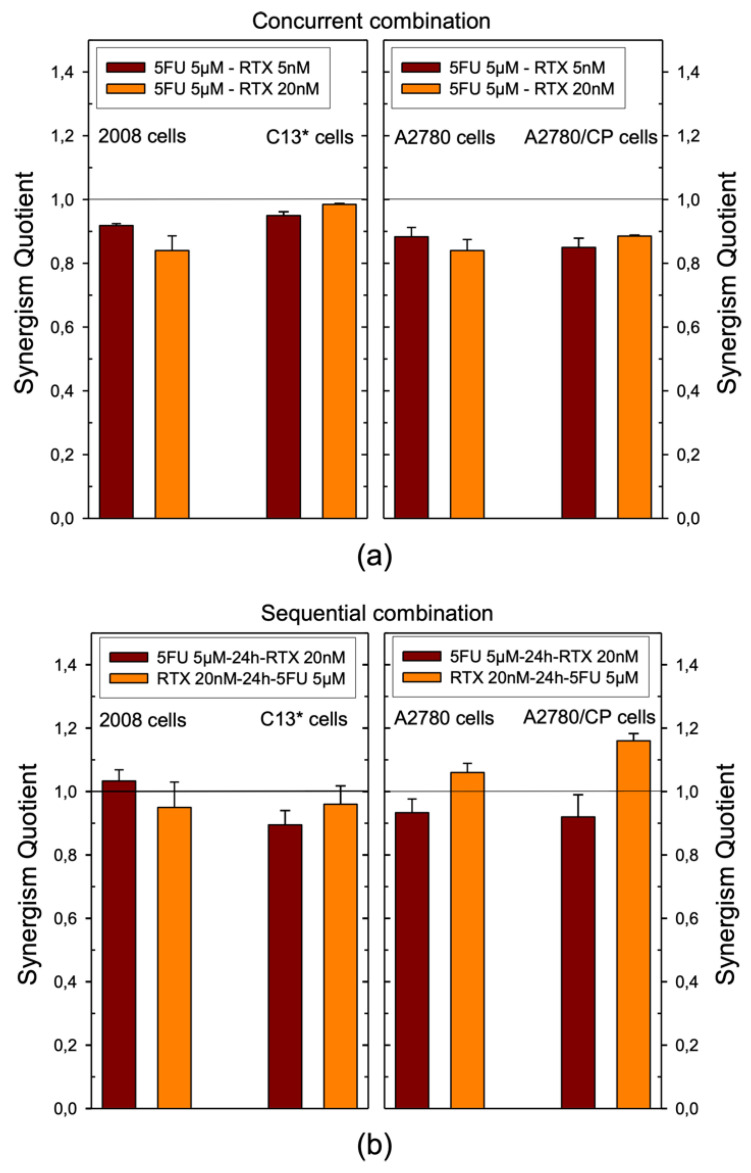
Synergism quotients for the inhibition of the growth of 2008, C13*, A2780, and A2780/CP cell lines caused by simultaneous (**a**) and sequential (**b**) administration of combinations of RTX and 5FU. Cell growth analysis was performed after exposing cells according to three schedules: simultaneous treatment (5FU/RTX), 5FU first, RTX 24 h later (5FU-24h-RTX), and the opposite (RTX-24h-5FU). The bars represent the mean of duplicate cell counts on three separate experiments. A quotient > 1 indicates synergism, =1, additivity <1 antagonism. Error bars represent SEM.

We have investigated the inhibitory effects of combinations of RTX and 5FU, with varying administration schedules, on the growth of four human ovarian cancer cell lines, 2008, C13*, A2780, and A2780/CP. These cells were either simultaneously exposed to RTX and 5FU for 72 h, or sequentially exposed to RTX for 24 h and then to RTX and 5FU for additional 48 h, or vice versa. The drug concentrations used in the combinations were based on the IC_50_s. These are reported in Table S3 of the Supplementary Information. The effects of binary combinations were determined with both drug concentrations lower than their IC_50_s and were characterized in terms of the synergism quotient [26,27,28]. The best results were achieved with 1:1000 or 1:250 RTX:5FU ratios, with 5 μM 5FU and either 5 or 20 nM RTX. With 5 μM 5FU alone, cell growth inhibitions were 24.7, 21.2, 28.3, and 27.1% for 2008, C13*, A2780, and A2780/CP, respectively. Analogously, 5 and 20 nM RTX alone inhibited the growth of these cells by 32.9 and 36, 26.4 and 31.2, 26.0 and 29.1, 24.5 and 25.2%, respectively (Table S4). The experiments indicated that the cytotoxicity of RTX and 5FU combinations were schedule dependent. In most cases, a simultaneous exposure to RTX and 5FU showed sub-additive or slightly antagonistic effects on all four cell lines, with synergism quotient (*sq*) values between 0.84 and 0.98. C13* cells were almost additively killed by 5µM 5FU simultaneously administered with 5 and 20 nM RTX: *sq* = 0.95 and 0.98 (Figure 6a, Table S4).

The RTX:5FU 1:1000 (5 nM:5 µM) concentration ratio produced antagonistic effects with both administration sequences (data not shown). On the contrary, when the two drugs were combined at a 1:250 ratio, 20 nM RTX:5 µM 5FU, the results on all cell lines were mostly additive or supra-additive. In particular, sequential RTX–5FU exposure produced *sq* values around 1 both on 2008 and C13* cells, but larger than 1, thus synergistic, on A2780 and A2780/CP cells, killing about 60% of cells (Figure 6b, Table S4).

**Figure 7:**
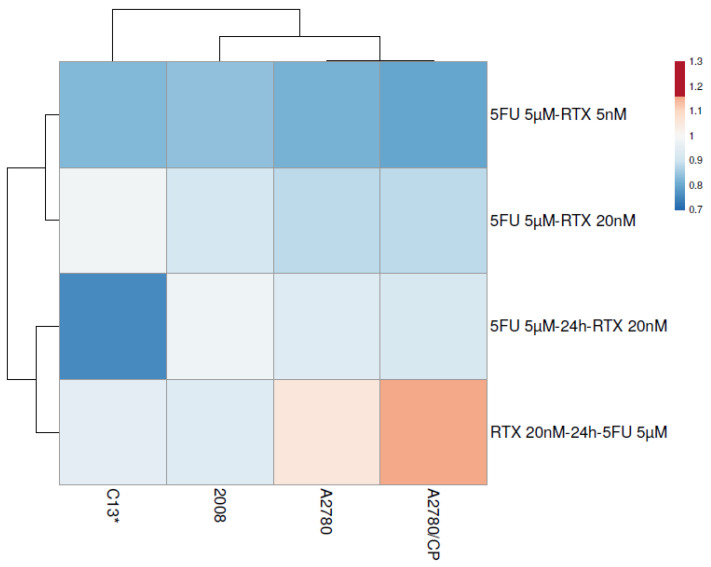
Heatmap representation of the synergism quotient values of the tested RTX-5FU combinations (rows) against the different cell lines (columns). Color code: red: *sq* > 1; blue: *sq* < 1. The data are reported in Table S4 of the Supplementary Information.

Indeed, the only orange/dark orange spots in the heatmap representation of the *sq* values shown in Figure 7 correspond to the additive/supra-additive combination of 20 nM RTX followed by 5 μM 5FU on A2780 and A2780/CP cells.

To investigate whether the observed effects of drug combinations on cell growth might be related to the cell cycle phase perturbations, the effects of RTX and 5FU on the cell cycle kinetics of five cell lines, 2008, C13*, A2780, A2780/CP, and IGROV-1, were assessed either with each drug alone or with their combination according to the three different administration sequences. Treatment of the A2780 cells with 5FU alone and in combination with RTX caused a slightly enhanced block in the G0/G1 phase of the cell cycle, from 57.3% in control untreated cells to values between 61.1 and 67.9% in treated cells.

**Figure 8:**
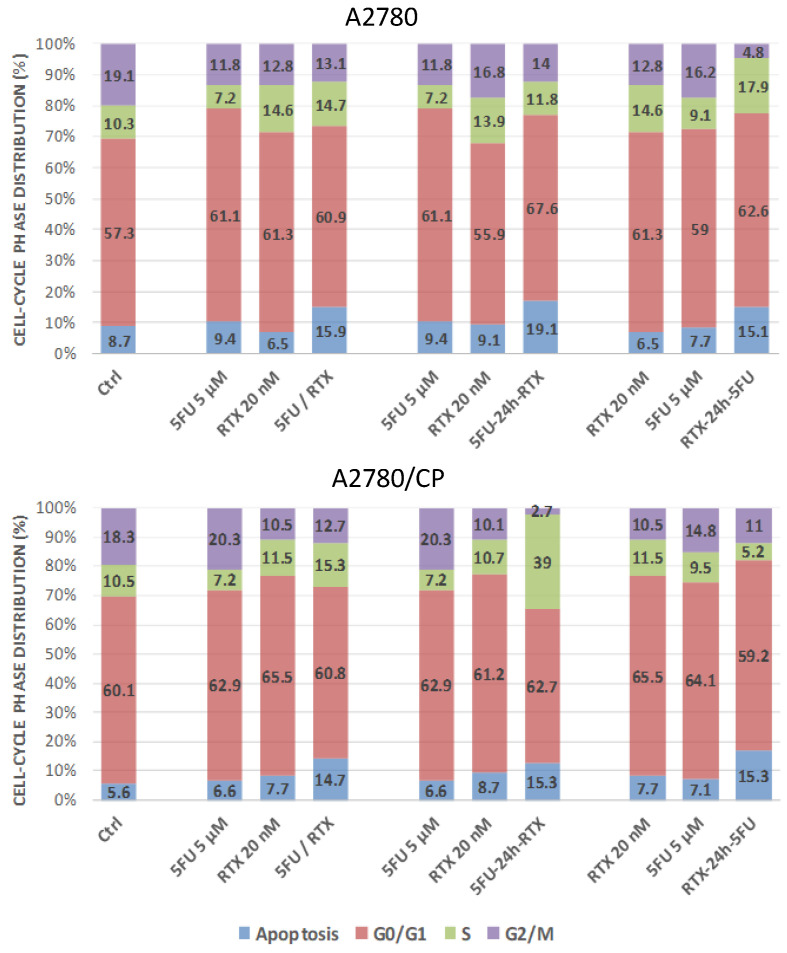
Cell cycle phase distribution percentages in cisplatin sensitive (A2780, upper panel) and resistant (A2780/CP, bottom panel) cancer cells treated with 5FU-RTX combinations with different combination patterns. Cell cycle phase distributions of A2780 and A2780/CP cells were determined by cytofluorimetric analysis of the PI-stained DNA content. Cells were exposed to 20 nM RTX, 5 μM 5FU alone, or with their combinations according to three schedules: simultaneous treatment (5FU/RTX) (left columns), 5FU first, RTX 24 h later (5FU-24h-RTX) (middle columns), and the opposite (RTX-24h-5FU) (right columns). Cells were treated with concentrations of each drug corresponding to 1:250 RTX:5FU ratio. Inserted numbers indicate the percentage of cells in the different phases of the cell cycle. The values are the mean of two/three experiments.

A concomitant decrease of cell accumulation in the G2/M phase was observed, in particular when RTX was added first, from 19.1% in control cells to 4.8% in treated ones. Together with this decrease, we observed a slightly larger cell accumulation in the S phase, from 10.3 to 17.9% (Figure 8 and Table S5 of the Supplementary Information). On the contrary, in the resistant A2780/CP cells, this effect was observed with the 5FU-24h-RTX sequence, with a remarkable cell block (38%) in the S phase. Both sensitive and resistant cells responded to the three treatment schedules with an additively enhanced percentage of apoptosis in comparison to each drug alone, in particular with both sequential treatments, reaching 15% and 19% of hypodiploid cells (Figure 8 and Table S5). On the contrary, treatment of the 2008, C13*, and IGROV-1 cell lines caused no major changes of the distribution in the different cell cycle phases compared with the control samples (Figure S6 and Table S6 of the Supplementary Information).

We can conclude that additive cell cycle perturbation can be observed in the case of A2780 cells treated with the sequential schedule with RTX administered first. This was more evident in sensitive (A2780) than in resistant cells (A2780/CP) where the opposite administration sequence was more effective in causing the S phase block.

## 4. Discussion

If considered together, all the described X-ray structures indicate that either the substrate or the cofactor can be replaced by an inhibitor, leaving space for the corresponding substrate/cofactor molecule or a second inhibitor. However, we have shown that for combinations of FdUMP and RTX to give synergistic inhibition of hTS the two inhibitors must bind the enzyme together to form the hTS:FdUMP:RTX ternary complex. A comparative analysis of the X-ray structure of the hTS:FdUMP:RTX ternary complex, reported here for the first time, with the X-ray structures of other binary or ternary complexes involving TS and its substrates or substrate analogs may help identify the determinants of the stability of this complex, thus translating them into mechanistic information.

The available structures include three ternary complexes of hTS with dUMP and RTX (PDB: 1HVY [15], 1I00 [32], 5X5Q [35]) as well as the corresponding ternary complexes in which RTX is replaced by methotrexate (MTX, PDB: 5X66 [35]) or an RTX analog (PDB: 5X67 [35]). They are all very similar, the main difference is in the hTS:dUMP:RTX ternary complex (PDB: 1HVY), in which dUMP is covalently bound to the catalytic Cys195 [15].

FdUMP is covalently bound to the catalytic cysteine in some ternary complexes of nonhuman TSs with THF or mTHF such as bacterial [39,40,41] and mouse source [37] Overall, these observations suggest that it is the presence of the reduced cofactor (mTHF) or an analog (THF), rather than of a folate-like inhibitor that favors formation of a covalent bond between dUMP and TSs. Therefore, it is reasonable that our ternary complex does not show a covalent link to the protein.

The mechanism of hTS inhibition by FdUMP alone involves the Michael addition of the thiol group of Cys195 to the C6 of the uracil ring [42]. This is consistent with the tens-of nM range for the K_d_ of the hTS:FdUMP binary complex that we have estimated (see Section 3.2), and suggests that FdUMP may work as a firmly bound partner in the formation of a stable ternary complex with the mTHF cofactor or the folate-like inhibitors. However, no covalent bond between the protein and the nucleotide is found in the X-ray crystal structure of the hTS:FdUMP:RTX ternary complex. In order for the latter to be thermodynamically competitive with the single inhibitor hTS:FdUMP:mTHF and hTS:dUMP:RTX complexes, which is a condition for the two inhibitors to act synergistically, RTX and FdUMP must contribute an extra-stabilization to the dual-inhibitor ternary complex. Indeed, as shown in Figure 2, stacking interactions occur between the FdUMP pyrimidine ring and the quinazoline ring of RTX and contribute to the stability of the complex. This is further stabilized by the strong non-covalent interaction between fluorine and the Trp109 side chain.

The end point of our crystal structure analysis is that a stronger inhibition of hTS is possible when both inhibitors are bound and may produce a synergistic combination effect on the protein. The possibility to translate this structural/kinetic correlation into a biological effect of the same sign was among the results of the cellular growth inhibition experiments performed on different ovarian cancer cells. We found that a sequential administration of, first, RTX and then 5FU, that is transformed intracellularly to FdUMP, results in a synergistic or supra-additive effect on three out of four cell lines. In the case of resistant cancer cells (A2780/CP) this effect was also confirmed after cell cycle analysis.

The observed schedule-dependent synergism in cell growth inhibition parallels the results of other preclinical and clinical investigations [43] and may result from a variety of specific cellular metabolic processes that are activated after administration of the two drugs and affect their pharmacokinetics (PK). RTX is transported into cells via the folate transporter 1 (FOLT, also known as reduced folate carrier protein). Similarly to MTX, it undergoes rapid addition of a pentaglutamate chain by folylpolyglutamyl synthetase (FPGS) yielding RTX-Glu(5), a chemical modification that increases its TS inhibition potency by up to 100 times and greatly extends its intracellular retention and availability [28,44]. Antifolates, especially if polyglutamylated, were shown to favor binding of FdUMP to hTS [45]. In particular, while such a binding was favored by mTHF with a regularly increasing concentration dependence, pentaglutamylated RTX enhanced FdUMP binding to hTS with a concentration dependence that showed a maximum at 0.25 micromoles/liter and then declined slowly, i.e., at concentrations that, according to the authors, are comparable with those clinically achievable by these drugs.

Concerning the latter point, due to the complexity of the events occurring when drugs are administered to cells or to a human body, providing mechanistic explanations for the schedule dependence of drug combinations is very challenging, although recently developed software simulating drug-interaction networks have produced encouraging results in the prediction of synergistic effects in therapy [46,47,48].

The clinical regimen doses, typically 28–39 mg h/L for 5FU [43] and 2–3 mg/m^2^ per infusion for RTX [5], correspond to a stationary concentration of the former in plasma between 4.7 and 6.5 microMolar and a peak plasma concentration of the latter around 1.5 microMolar. In view of an estimated cytosolic concentration of TS of few hundreds of nanomoles/liter [49] and the nanomolar/tens of nanomolar values for the affinities of RTX and 5FdUMP reported in Section 3.2, such concentrations ensure full occupancy of the protein binding sites, thus producing synergistic TS inhibition by the two drugs according to the mechanistic results in Section 3.3.

Several additional contributions to the observed RTX-5FU synergistic activity on cell growth and, to some extent, its schedule dependence can be postulated based on evidence from the biological literature and are related to the PK of the two drugs. The first one comes from the ability of RTX to activate the catabolism of 5FU by inhibiting dihydropyrimidine dehydrogenase, thus drastically reducing the intracellular levels of the drug [43]. Combined with the long intracellular persistence of RTX after polyglutamylation [28,44], this induced catabolism of 5FU may contribute to the reported RTX-5FU synergy and explain its occurrence when RTX is administered first. Another contribution, also justifying the mentioned schedule dependence, might be related to the increase in the intracellular levels of phosphoribosylpyrophosphate (PRPP) observed following a 24 h exposure of colon carcinoma cells to RTX [6]. The increased levels of PRPP reduce the availability of nucleotides and therefore favor incorporation of FdUMP into RNA [44]. This in turn causes a reduction of the purine and DNA synthesis. Finally, RTX-5FU synergism might also result from a depletion in dTTP that occurs following TS inhibition by either agent and that may induce negative feedback on deoxycytidylate deaminase, ribonucleotide reductase, and thymidine kinase that synergistically block DNA synthesis [50].

We observed qualitatively different synergistic effects between sensitive and resistant cell lines (Figure 7). The difference may derive from their different expression of the enzymes of the folate cycle and nucleotide metabolism, which was shown to affect the cellular response to another antifolate, pemetrexed [23,51].

## 5. Conclusions

The hTS:FdUMP:RTX X-ray structure presented here provides evidence of a strong stabilization of the protein upon binding of both drugs. The existence of the attractive stacking interactions between the pyrimidine and quinazoline rings and the absence of hindering interactions between the two bound inhibitors lead to a stabilization of the active complex. Therefore, together with the analysis of the hTS-inhibition mechanistic pattern of the two drugs, the structural information forms a consistent picture of tangential cooperativeness between the two hTS inhibitors and represents a molecular indicator for their possible synergism in the treatment of ovarian cancers. As for the biological effects of the combined treatment with the two inhibitors, despite similar effects of the three sequences on cell cycle distribution of the four cell lines, the results of the combination analysis at the cellular level suggest that sequential administration of RTX followed by 5FU is synergistic and an efficient treatment sequence against human sensitive and resistant ovarian cancer cell growth.

Consideration about a direct link between the concentration adopted in the crystallographic experiments and its translation into the complexity of the drug behavior in the human body lead us to the end point of the biological activity of the drugs in vivo, i.e., pharmacodynamics. The basic condition for eliciting a synergistic drug response is that both drugs can simultaneously bind the TS target. Full occupancy of the target binding sites by both drugs is ensured, given their high affinities, if they are available at concentrations 3–5 times higher than the intracellular concentration of the TS target.

We have shown that inhibition kinetic studies on recombinant proteins and X-ray crystallography can disclose indicators of the cellular behavior of two drugs targeting the hTS protein. This strategy can be helpful in the exploration of novel drug combinations, also directed towards other targets. In general, knowledge of basic molecular, namely, structural, and mechanistic properties of two drugs can suggest, already in the early phase of preclinical drug research, the ability of combinations of the two drugs to inhibit cancer cell growth. Our work can provide relevant figures to feed recently developed dedicated software that can simulate drug-interaction networks and reasonably predict the therapeutic doses necessary for achieving a synergistic use of drug combinations. Thus, the provided basic experimental structural and kinetic/thermodynamic information on drug(s):protein complexes can translate into a drug combination therapeutic plan. The synergistic drugs combination, in particular are very important in anticancer therapy because, by reducing the relative amount of each drug, the drug toxicity is limited with benefit for the patient. In perspective, this work may have a rationalizing and time-saving impact on therapeutic applications.

## Data Availability

The Supplementary Materials are available at the following address: https://zenodo.org/deposit?page=1&size=20.

## Supplementary Materials

The following are available online at https://www.mdpi.com/article/10.3390/cancers13092061/s1, Table S1, Data collection, and processing statistics; Table S2. Structure solution and refinement statistics; Table S3, 5FU and RTX IC_50_ values; Table S4, Growth inhibition and synergism quotients; Table S5, Effects of 72 h-exposure to the drugs alone and in combination on A2780 and A2780/CP cell lines; Table S6, Effects of 72 h-exposure to the drugs alone and in combination on 2008, C13* and IGROV-1cell lines; Kinetic analysis for a single, tight-binding inhibitor; Figure S1, Solutions of the kinetic scheme; Figure S2, Synergy quotients (SQ) calculated assuming K_m_’ = K_m_ and K_M_’ = K_M_; Figure S3, Synergy quotients (SQ) calculated assuming K_m_’ = 0.5 K_m_ and K_M_’ = K_M_; Figure S4, Synergy quotients (SQ) calculated assuming K_m_’ = K_m_ and K_M_’ = 10 K_M_; Figure S5, Synergy quotients (SQ) calculated assuming K_m_’ = 0.5 K_m_ and K_M_’ = 10 K_M_; Figure S6, Effect of drug combinations on the cell cycle phase distribution of 2008, C13*, and IGROV-1 cell lines.

## Abbreviations

FdUMP: 5-fluoro-2′-deoxyuridine 5′-monophosphate; 5FU: 5-fluorouracil; RTX: N-[(5-{methyl[(2-methyl-4-oxo-1,4-dihydroquinazolin-6-yl)methyl]amino}-2-thienyl)carbonyl]-L-glutamic acid; dUMP: 2′-deoxyuridine-5′- monophosphate; mTHF: N^5^,N^10^-methylenetetrahydrofolate; β-ME: β-mercaptoethanol.
