# Supplementary material for: Structural Bases for the Synergistic Inhibition of Human Thymidylate Synthase and Ovarian Cancer Cell Growth by Drug Combinations
Source: Cancers (Basel). 2021 Apr 24;13(9):2061. doi: 10.3390/cancers13092061 (PMC8123127; doi:10.3390/cancers13092061)
Supplement: Supplementary file 1 [file cancers-13-02061-s001.zip › cancers-1170867-supplementary.pdf]

SUPPLEMENTARY INFORMATION

Structural bases for the synergistic inhibition of human thymidylate synthase and cancer cell growth by drugs combinations.

Cecilia Pozzi[1^], Matteo Santucci[2^], Gaetano Marverti[3], Domenico D'Arca[3], Lorenzo Tagliazucchi[2], Stefania Ferrari[2], Gaia Gozzi[2], Lorena Losi[2], Giusi Tassone[1], Stefano Mangani[1], Glauco Ponterini[2*], Maria Paola Costi[2*]

[1] Department of Biotechnology, Chemistry and Pharmacy, Department of Excellence 2018-2022, University of Siena, Via A. Moro 2, 53100 Siena, Italy; pozzi4@unisi.it; giusy.tassone@unisi.it; stefano.mangani@uinsi.it

[2] Department of Life Sciences, University of Modena and Reggio Emilia, Via G. Campi 103, 41125, Modena, Italy; matteo.santucci86@gmail.com; lorenzotagliazucchi@unimore.it; sferrari591@gmail.com; g.gozzi@holostem.com; lorena.losi@unimore.it; glauco.ponterini@unimore.it;mariapaola.costi@unimore.it.

[3] Department of Biomedical, Metabolic and Neural Sciences, Via G. Campi 287, University of Modena and Reggio Emilia, 41125 Modena, Italy; gaetano.marverti@unimore.it; domenico.darca@unimore.it;

* Correspondence: glauco.ponterini@unimore.it (G.P.); mariapaola.costi@unimore.it (M.P.C).

^ These authors contributed equally to this work.

Table of contents:

Table S1. S2

Table S2. S3

Table S3. S4

Table S4. S5

Table S5. S7

Table S6. S8

Kinetic analysis for a single, tight-binding inhibitor S9

Figure S1. S11

Figure S2. S12

Figure S3. S13

Figure S4. S13

Figure S5. S14

Figure S6. S15

Table S1. IC_{50} values (μM or nM ± SD) for selected compounds obtained after 72 h treatment of 2008, C13*, A2780 and A2780/CP human ovarian cancer cells. Data indicate mean values and standard deviation from three experiments performed in duplicate.

Compound	2008 cells	C13* cells	A2780 cells	A2780/CP cells
**5-FU (μM)**	6.3±0.4	12.4±0.8	9.5±0.8	12.9±1.1
**RTX (nM)**	9.5±1	26.2±3	8.6±1.2	31.1±2.9

Table S2. Growth inhibition caused by simultaneous or sequential drug administration on 2008, C13*, A2780 and A2780/CP cell lines, and corresponding synergism quotients. The cells were treated with the drugs alone or their combinations at days 1 and 2, and were counted at day 4.

Drug combination	% Growth inhibition[a]	Synergism quotient	% Growth inhibition[a]	Synergism quotient
	**2008 cells**		**C13* cells**	
**5FU 5µM/RTX 5nM**	55.3 / (24.7+36.0)	0.91	45.5 / (21.2+26.4)	0.95
**5FU 5µM/RTX 20nM**	49.1 / (24.7+32.9)	0.85	51.2 / (21.2+31.2)	0.98
**5FU 5µM-24h-RTX 20nM**	57.6 / (24.7+30.2)	1.05	48.2 / (21.2+32.4)	0.90
**RTX 20nM-24h-5FU 5µM**	55.2 / (36.0+22.1)	0.95	51.3 / (31.2+22.3)	0.96
	**A2780 cells**		**A2780/CP cells**	
**5FU 5µM/RTX 5nM**	49.4 / (28.3+27.0)	0.89	44.2 / (27.1+24.5)	0.85
**5FU 5µM/RTX 20nM**	48.5 / (28.3+29.1)	0.84	45.7 / (27.1+25.2)	0.87
**5FU 5µM-24h-RTX 20nM**	51.4 / (28.3+26.6)	0.93	45.8 / (27.1+22.5)	0.92
**RTX 20nM-24h-5FU 5µM**	58.8 / (29.1+27.3)	1.05	60.4 / (25.2+26.8)	1.16

[a] Data are expressed as average percentage values of growth inhibition relative to the growth of untreated control cells of duplicate cell counts on three separate experiments and indicate the inhibition by the drug combination divided by the sum of the inhibitions by the single drugs to obtain the synergism quotients (*SQ*). A quotient larger than unity indicates a synergistic effect, while a quotient lower than unity indicates an antagonistic effect.

Table S3. Effects of 72h-exposure to the drugs alone and in combination on the cell cycle phase distribution of A2780 and A2780/CP cell lines determined by cytofluorimetric analysis. The reported values are the means of two/three experiments.

A2780 cells

	Apoptosis	G0/G1	S	G2/M
**Ctrl**	8.7±0.8	57.3±6.1	10.3±0.9	19.1±2.2
**5FU 5µM**	9.4±1.6	61.1±2.1	7.2±1.9	11.8±4.0
**RTX 20nM**	6.5±2.4	61.3±5.5	14.6±0.5	12.8±3.9
**5FU/RTX**	15.9±2.8	60.9±7.6	14.7±1.8	13.1±0.8
**5FU 5µM**	9.4±1.6	61.1±2.1	7.2±1.9	11.8±4.0
**RTX 20nM**	9.1±0.9	55.9±7.1	13.9±1.8	16.8±3.3
**5FU-24h-RTX**	19.1±1.4	67.6±8.8	11.8±2.2	14.1±2.0
**RTX 20nM**	6.5±2.4	61.3±5.5	14.6±0.5	12.8±3.9
**5FU 5µM**	7.7±1.3	59.1±5.8	9.1±1.1	16.2±3.1
**RTX-24h-5FU**	15.1±2.0	62.6±7.8	17.9±1.8	4.8±0.9

A2780/CP cells

	Apoptosis	G0/G1	S	G2/M
**Ctrl**	5.6±0.6	60.1±6.8	10.5±2.0	18.3±1.9
**5FU 5µM**	6.6±0.5	62.9±1.1	7.2±2.3	20.3±5.5
**RTX 20nM**	7.7±2.0	65.5±2.3	11.5±1.2	10.5±1.4
**5FU/RTX**	14.7±1.8	60.8±4.2	15.3±1.7	12.7±1.7
**5FU 5µM**	6.6±0.5	62.9±1.1	7.2±2.3	20.3±5.5
**RTX 20nM**	9.7±2.8	62.2±4.1	12.7±1.5	9.1±0.8
**5FU-24h-RTX**	15.3±2.2	62.7±3.8	39.0±2.9	2.7±0.5
**RTX 20nM**	7.7±2.0	65.5±2.3	11.5±1.2	10.5±1.4
**5FU 5µM**	7.1±0.9	64.1±5.8	9.5±2.3	14.8±2.0
**RTX-24h-5FU**	15.3±1.7	59.2±6.1	5.2±0.4	11.0±0.5

Cytofluorimetric analysis. 24 h after seeding, the cells were exposed to the drugs, then the DNA content of untreated and treated cells was determined by flow cytometry after propidium iodide staining. Because of nuclear fragmentation, apoptotic cells are characterized by a lower DNA content (hypodiploid cells, having fewer chromosomes than the diploid cells number of chromosomes).

Table S4. Same as in Table S3 for 2008, C13* and IGROV-1cell lines.

2008 cells

	Apoptosis	G0/G1	S	G2/M
**Ctrl**	6.7±0.6	62.3±6.6	9.3±0.5	15.9±1.6
**5FU 5µM**	15.5±2.1	51.7±5.6	11.5±0.9	10.8±0.4
**RTX 20nM**	16.7±1.6	67.7±4.4	9.9±0.8	9.8±1.1
**5FU/RTX**	20.6±2.7	60.2±6.0	13.8±2.1	9.6±0.9
**5FU 5µM**	15.5±2.1	51.7±5.6	11.5±0.9	10.8±0.4
**RTX 20nM**	13.1±1.1	60.3±2.9	10.8±0.8	13.7±1.3
**5FU-24h-RTX**	23.8±3.6	56.9±6.1	11.4±0.6	7.9±0.3
**RTX 20nM**	16.7±1.6	67.7±4.4	9.9±0.8	9.8±1.1
**5FU 5µM**	16.1±0.9	60.5±3.9	12.5±1.6	13.1±0.8
**RTX-24h-5FU**	18.8±2.3	58.6±6.2	11.2±0.5	8.8±0.3

C13* cells

	Apoptosis	G0/G1	S	G2/M
**Ctrl**	10.7±0.3	54.5±4.4	13.2±2.3	13.8±0.5
**5FU 5µM**	16.6±0.4	59.6±1.6	9.2±1.0	8.6±0.3
**RTX 20nM**	11.7±0.4	58.8±1.1	13.7±0.5	12.9±1.2
**5FU/RTX**	25.0±2.5	52.0±5.3	7.9±3.3	8.2±2.1
**5FU 5µM**	16.6±0.4	59.6±1.6	9.2±1.0	8.6±0.3
**RTX 20nM**	14.7±2.5	57.9±5.3	13.5±2.1	9.5±2.5
**5FU-24h-RTX**	21.4±2.8	62.3±6.6	10.9±2.0	4.3±0.9
**RTX 20nM**	11.7±0.4	58.8±1.1	13.7±0.5	12.9±1.2
**5FU 5µM**	15.7±1.1	46.0±2.9	18.5±2.2	11.2±1.9
**RTX-24h-5FU**	19.8±2.3	46.4±4.8	15.5±2.6	11.7±2.2

IGROV-1 cells

	Apoptosis	G0/G1	S	G2/M
**Ctrl**	0.87±0.4	74.6±7.3	8.41±2.0	16.5±3.3
**5FU 5µM**	1.1±0.2	77.4±6.1	9.1±1.3	12.8±2.8
**RTX 20nM**	2.8±1.2	70.2±3.4	13.1±1.8	11.4±2.7
**5FU/RTX**	2.2±0.3	75.5±5.3	11.4±0.9	11.1±1.2
**5FU 5µM**	1.1±0.2	77.4±6.1	9.1±1.3	12.8±2.8
**RTX 20nM**	1.3±0.5	73.3±6.5	10.8±2.1	13.8±2.2
**5FU-24h-RTX**	1.9±0.2	77.5±8.3	9.3±0.5	11.5±2.0
**RTX 20nM**	2.8±1.2	70.2±3.4	13.1±1.8	11.4±2.7
**5FU 5µM**	0.9±0.3	72.7±4.3	13.9±2.1	12.8±2.9
**RTX-24h-5FU**	1.7±0.4	71.7±5.6	15.2±2.1	10.6±1.3

Table S5. Data collection and processing. Values for the outer shell are given in parentheses.

	hTS:FdUMP:RTX ternary complex
PDB code	6ZXO
Diffraction source	ESRF ID23-2
Wavelength (Å)	0.8726
Temperature (K)	100
Detector	MarMOSAIC 225 mm CCD
Crystal-detector distance (mm)	265.84
Rotation range per image (°)	0.5
Total rotation range (°)	180
Exposure time per image (s)	2.5
Space group	P1
No. of subunit in asymmetric unit	6 (3 dimers)
Cell parameters (Å, °)	
a	61.72
b	95.87
c	103.84
α	112.39
β	91.47
γ	109.17
Mosaicity (°)	1.20
Resolution range (Å)	58.15 – 2.60 (2.74-2.60)
Total reflections	117483 (16977)
Unique reflections	60436 (8757)
Completeness (%)	96.0 (95.7)
R_{meas}	0.110 (0.518)
I/σ(I)	6.5 (2.0)
Multiplicity	1.9 (1.9)
Overall *B* factor from Wilson plot (Å^2)	48.2

Table S6. Structure solution and refinement. Values for the outer shell are given in parentheses.

	hTS:FdUMP:RTX ternary complex
PDB code	6ZXO
Resolution range (Å)	58.15 – 2.60 (2.67-2.60)
Completeness (%)	96.0 (95.7)
No. of reflections, working set	57340 (3084)
No. of reflections, test set	4227 (222)
R_{cryst}	17.2 (32.2)
R_{free}	23.2 (33.3)
No. of non-H atoms	
Protein	13609
Ligands (FdUMP, RTX)	318 (126, 192)
Water	376
Ethylene glycol	12
Total	14315
Average B factors (Å^2)	51.6
Protein	52.9
Ligands (FdUMP, RTX)	52.4 (41.9, 59.3)
Water	49.5
Ethylene glycol	65.5
R.m.s. deviations	
bond lengths (Å)	0.006
bond angles (°)	1.611
chiral volumes (Å^3)	0.116
planes (Å)	0.006
Estimate error on coordinates based on R value (Å)	0.26
Ramachandran plot	
Most favored (%)	94.6
Allowed (%)	5.4
Not allowed (%)	0.0
RSCC	
FdUMP chain A, B, C, D, E, F	0.99, 0.99, 0.99, 0.98, 0.98, 0.98
RTX chain A, B, C, D, E, F	0.96, 0.95, 0.93, 0.96, 0.93, 0.96

Kinetic analysis for a single, tight-binding inhibitor

The kinetic scheme below (the same as in Figure 4a) accounts for the sequential binding of dUMP (D) and mTHF (M) to hTS (E) and inhibition by the substrate analogue, FdUMP (d), and the cofactor analogue, RTX (m).

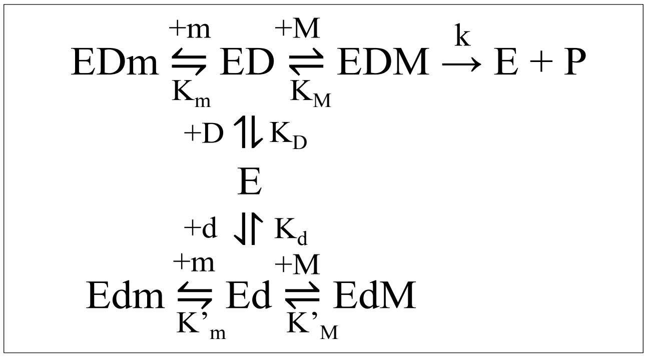

Following a standard approach for the inhibition analysis in the case of a tight-binding ligand (ref. I.H.Segel, Enzyme Kinetics, Wiley, 1993, New York, ch. 3E) that only assumes fast equilibration, we have obtained the equations below for the inhibitions by FdUMP (d) alone (eq. S1) and RTX (m) alone (eq. S2).

$$L_{FdUMP} = \frac{d_t(1+\frac{M}{K'_M})}{v_{max}-v(1+\frac{K_M}{M}+\frac{K_D K_M}{D\,M})} = \frac{1}{k}\left(1+\frac{M}{K'_M}\right)+\frac{D\cdot M K_d}{K_D K_M}\frac{1}{v} \quad \text{(S1)}$$

$$L_{RTX} = \frac{m_t}{v_{max}-v(1+\frac{K_M}{M}+\frac{K_D K_M}{D\cdot M})} = \frac{1}{k}+\frac{M K_m}{K_M}\frac{1}{v} \quad \text{(S2)}$$

Here, v is the reaction rate, m, d, M and D represent the molar concentrations of the corresponding species and t means total.

In kinetic experiments performed at constant substrate and cofactor concentrations and variable inhibitor concentration, the measured rates can be combined with known v_{max} and K_M and K_D values to compute the left members, L. These are plotted versus v^{-1}. After a linear least-square analysis, we can check the consistency of our treatment by comparing the k value obtained from the intercept with the known k value. On the other hand, from the slope and the known K_M, K_D, M and D values, we obtain K_d and K_m. In the case of FdUMP, the slope, hence K_d, depend on the value chosen for K_M', the

equilibrium constant for dissociation of M from EdM, in the calculation of L_{FdUMP}. Instead, k turns out to be independent on this choice (see the synergistic inhibition paragraph in the main text).

$$v_{ni} = \frac{v_{max}}{1+\frac{K_M}{M}+\frac{K_D K_M}{D\cdot M}}$$

$$v_m = \frac{v_{max}}{1+\frac{K_M}{M}(1+\frac{m}{K_m})+\frac{K_D K_M}{D\cdot M}} \qquad v_d = \frac{v_{max}}{1+\frac{K_M}{M}(1+\frac{K_D}{D}+\frac{K_D}{D}\frac{d}{K_d}+\frac{K_D\, d\cdot M}{D K_d K'_M})}$$

$$v_{dm} = \frac{v_{max}}{1+\frac{K_M}{M}(1+\frac{m}{K_m})+\frac{K_D K_M}{D\cdot M}(1+\frac{d}{K_d}+\frac{d\cdot m}{K_d K'_m}+\frac{d\cdot M}{K_d K'_M})}$$

$$sq = \frac{\frac{1}{A+B}-\frac{1}{A+\frac{K_M m}{M K_m}+B(1+\frac{d}{K_d}+\frac{d\cdot m}{K_d K'_m}+\frac{d\cdot M}{K_d K'_M})}}{\frac{2}{A+B}-\frac{1}{A+\frac{K_M m}{M K_m}+B}-\frac{1}{A+B(1+\frac{d}{K_d}+\frac{dM}{K_d K'_M})}}$$

$$A = 1+\frac{K_M}{M} \qquad B = \frac{K_D K_M}{D\cdot M}$$

Figure S1. Solutions of the kinetic scheme obtained under the assumptions of Michaelis-Menten enzyme kinetics. v_{ni} is the reaction rate in the absence of inhibitors, v_d and v_m are the reaction rates in the presence of each of the inhibitors alone and v_{dm} in the presence of the two inhibitors together; sq is the synergism quotient, defined as $sq = (v_{ni} - v_{dm})/(2v_{ni} - v_d - v_m)$, expressed in terms of the concentrations of the substrates (D = [dUMP] and M = [mTHF]) and of the inhibitors (d = [FdUMP] and m = [RTX]) and of the dissociation constants of the equilibria they are involved in.

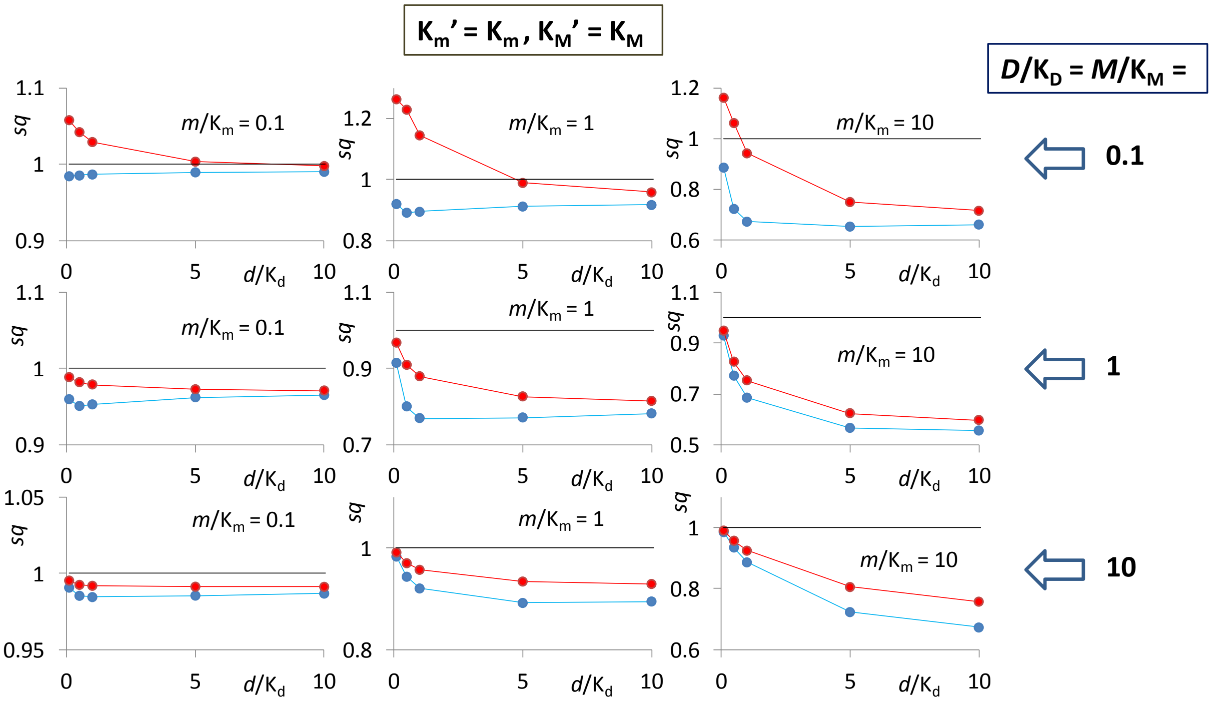

Figure S2. Synergy quotients (*sq*) calculated according to the equation in Figure S1 and derived from the solution of the kinetic scheme in Figures 3 and S1 for several values of the ratios of inhibitor concentrations (*m* and *d*) to their corresponding dissociation constants ($K_{m,d}$). The ratios of the concentrations of the substrate (*D*) and the cofactor (*M*) to the corresponding dissociation constants (see Figure 2) are, respectively, $D/K_D = M/K_M = 0.1$ for the three plots in the upper row, $D/K_D = M/K_M = 1$ in the central row and $D/K_D = M/K_M = 10$ in the lower row. The red symbols represent solutions obtained by allowing the two inhibitors to bind the enzyme simultaneously (Edm complex in the kinetic scheme); alternatively, the case of mutual exclusion of the two inhibitors gives results represented by the blue symbols. Assumptions: $K_m' = K_m$ and $K_M' = K_M$.

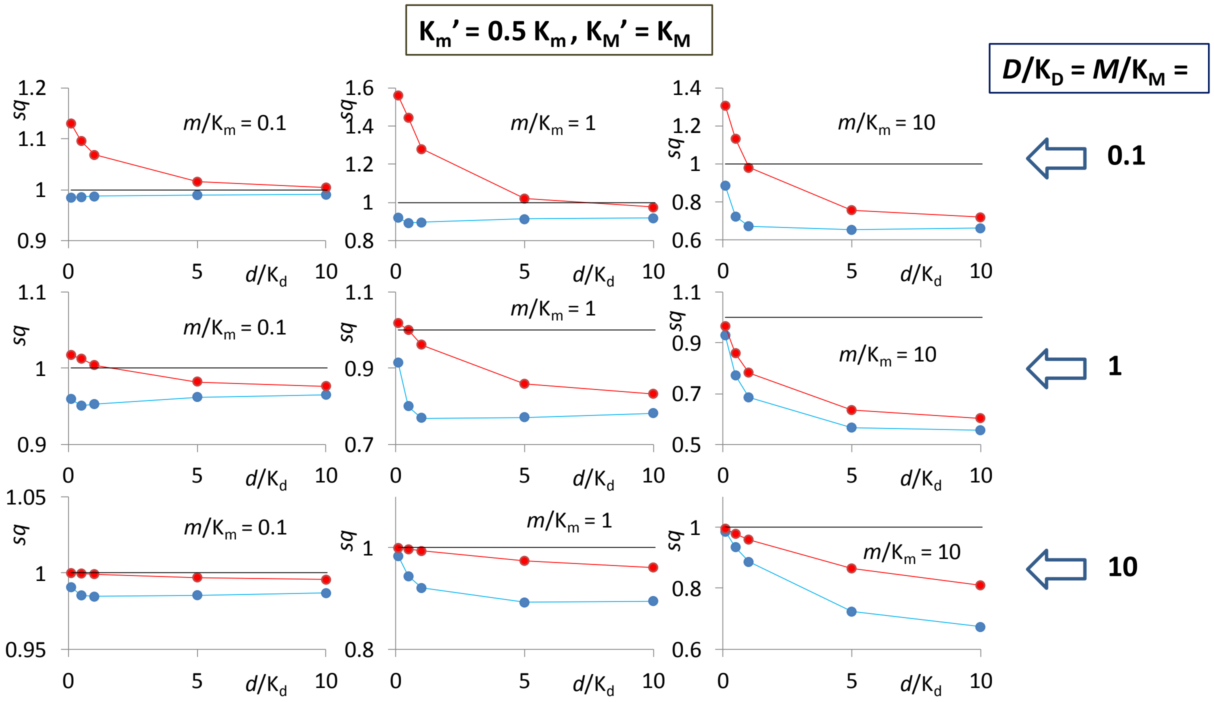

Figure S3. Same as in Fig. S2 but assuming $K_m' = 0.5\ K_m$ and $K_M' = K_M$.

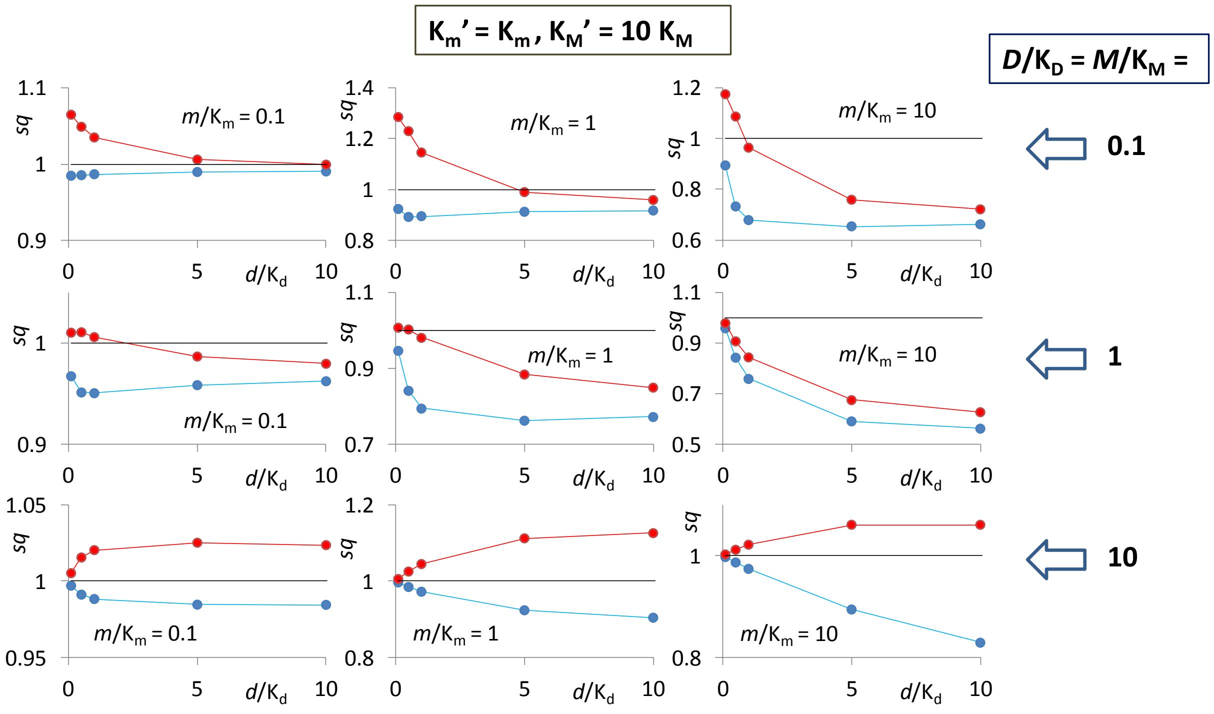

Figure S4. Same as in Fig. S2, but assuming $K_m' = K_m$ and $K_M' = 10\ K_M$.

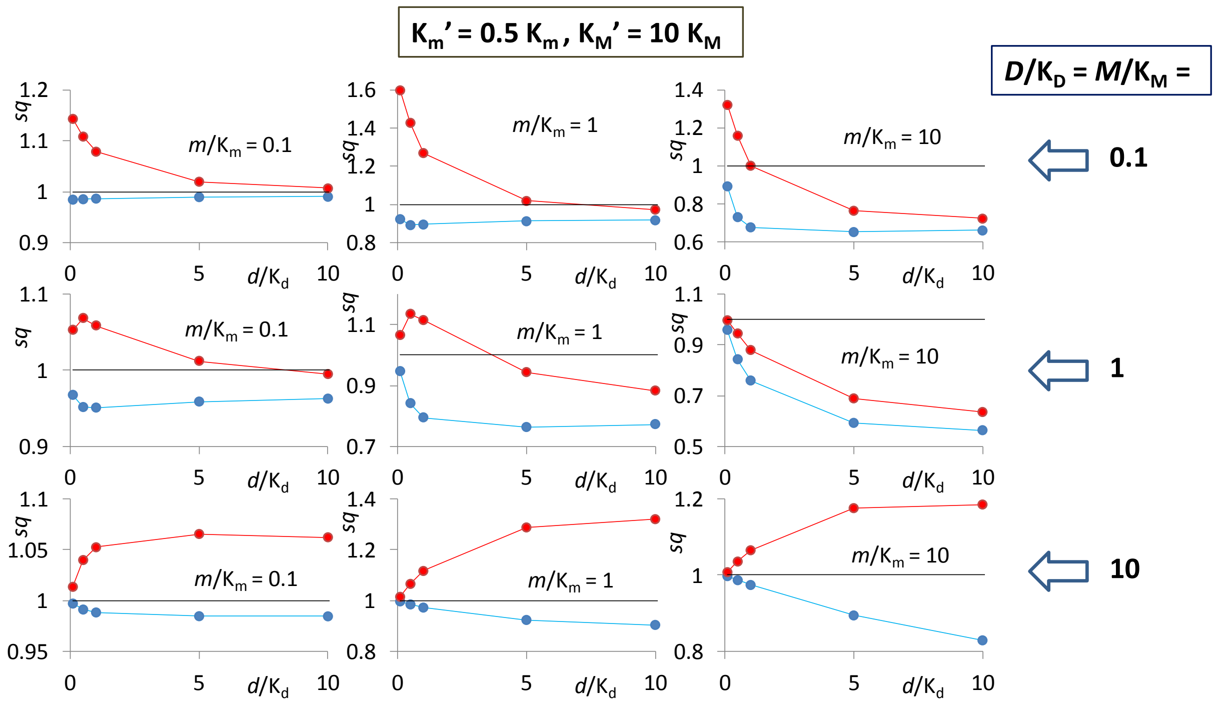

Figure S5. Same as in Fig. S2, but assuming $K_m' = 0.5\ K_m$ and $K_M' = 10\ K_M$.

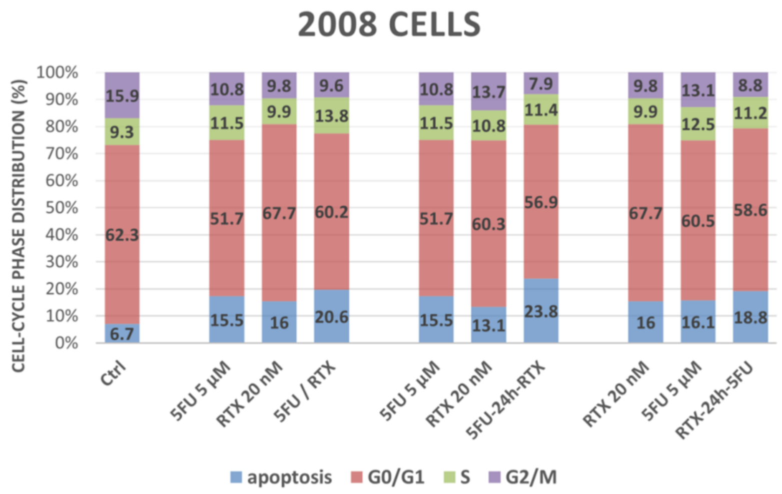
2008 CELLS
CELL-CYCLE PHASE DISTRIBUTION (%)
100%
90%
80%
70%
60%
50%
40%
30%
20%
10%
0%
Ctrl
5FU 5 µM
RTX 20 nM
5FU / RTX
5FU 5 µM
RTX 20 nM
5FU-24h-RTX
RTX 20 nM
5FU 5 µM
RTX-24h-5FU
6.7
62.3
9.3
15.9
15.5
51.7
11.5
10.8
16
67.7
9.9
9.8
20.6
60.2
13.8
9.6
15.5
51.7
11.5
10.8
13.1
60.3
10.8
13.7
23.8
56.9
11.4
7.9
16
67.7
9.9
9.8
16.1
60.5
12.5
13.1
18.8
58.6
11.2
8.8
apoptosis
G0/G1
S
G2/M

(a)

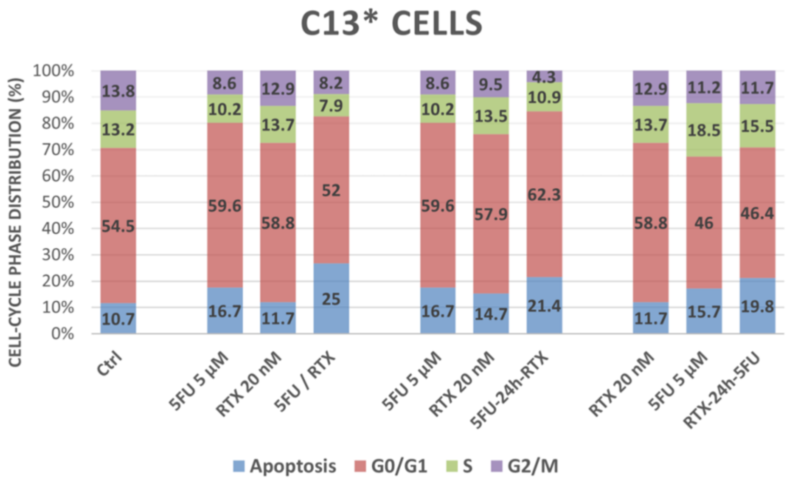
C13* CELLS
CELL-CYCLE PHASE DISTRIBUTION (%)
100%
90%
80%
70%
60%
50%
40%
30%
20%
10%
0%
Ctrl
5FU 5 µM
RTX 20 nM
5FU / RTX
5FU 5 µM
RTX 20 nM
5FU-24h-RTX
RTX 20 nM
5FU 5 µM
RTX-24h-5FU
10.7
54.5
13.2
13.8
16.7
59.6
10.2
8.6
11.7
58.8
13.7
12.9
25
52
7.9
8.2
16.7
59.6
10.2
8.6
14.7
57.9
13.5
9.5
21.4
62.3
10.9
4.3
11.7
58.8
13.7
12.9
15.7
46
18.5
11.2
19.8
46.4
15.5
11.7
Apoptosis
G0/G1
S
G2/M

(b)

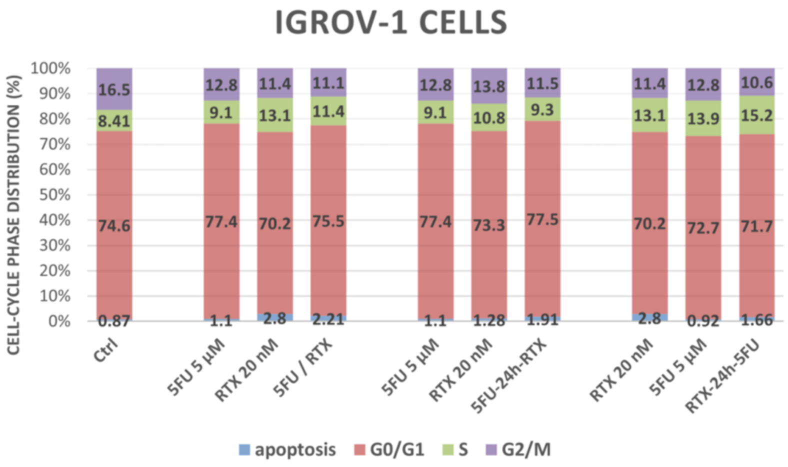
IGROV-1 CELLS
CELL-CYCLE PHASE DISTRIBUTION (%)
100%
90%
80%
70%
60%
50%
40%
30%
20%
10%
0%
Ctrl
5FU 5 µM
RTX 20 nM
5FU / RTX
5FU 5 µM
RTX 20 nM
5FU-24h-RTX
RTX 20 nM
5FU 5 µM
RTX-24h-5FU
0.87
74.6
8.41
16.5
1.1
77.4
9.1
12.8
2.8
70.2
13.1
11.4
2.21
75.5
11.4
11.1
1.1
77.4
9.1
12.8
1.28
73.3
10.8
13.8
1.91
77.5
9.3
11.5
2.8
70.2
13.1
11.4
0.92
72.7
13.9
12.8
1.66
71.7
15.2
10.6
apoptosis
G0/G1
S
G2/M

(c)

Figure S6. Effect of drug combinations on the cell cycle phase distribution of 2008, C13* and IGROV-1 cell lines measured by cytofluorimetric analysis of the DNA content following propidium iodide staining. Cell cycle analysis was performed after exposing cells to 20 mM RTX and 5 mM 5FU alone or in combination according to three schedules: simultaneous treatment; 5FU followed by RTX; RTX followed by 5FU. The numbers are the percentages of cells in the different phases of the cell cycle. The values are the mean of three experiments.
